# Early childhood malnutrition impairs adult resting brain function using near-infrared spectroscopy

**DOI:** 10.3389/fnhum.2023.1287488

**Published:** 2024-01-17

**Authors:** Kassandra Roger, Phetsamone Vannasing, Julie Tremblay, Maria L. Bringas Vega, Cyralene P. Bryce, Arielle Rabinowitz, Pedro Antonio Valdes-Sosa, Janina R. Galler, Anne Gallagher

**Affiliations:** ^1^LION Lab, Sainte-Justine University Hospital Research Center, University of Montreal, Montreal, QC, Canada; ^2^MOE Key Lab for Neuroinformation, The Clinical Hospital of Chengdu Brain Science Institute, University of Electronic Science and Technology of China, Chengdu, China; ^3^Barbados Nutrition Study, Bridgetown, Barbados; ^4^Montreal Neurological Institute, McGill University, Montreal, QC, Canada; ^5^Division of Pediatric Gastroenterology and Nutrition, MassGeneral Hospital for Children, Boston, MA, United States

**Keywords:** protein-energy malnutrition, NIRS, resting-state, functional connectivity, graph theory

## Abstract

**Introduction:**

Early childhood malnutrition affects 200+ million children under 5 years of age worldwide and is associated with persistent cognitive, behavioral and psychiatric impairments in adulthood. However, very few studies have investigated the long-term effects of childhood protein-energy malnutrition (PEM) on brain function using a functional hemodynamic brain imaging technique.

**Objective and methods:**

This study aims to investigate functional brain network alterations using near infrared spectroscopy (NIRS) in adults, aged 45–51 years, from the Barbados Nutrition Study (BNS) who suffered from a single episode of malnutrition restricted to their first year of life (n = 26) and controls (n = 29). A total of 55 individuals from the BNS cohort underwent NIRS recording at rest.

**Results and discussion:**

Using functional connectivity and permutation analysis, we found patterns of increased Pearson’s correlation with a specific vulnerability of the frontal cortex in the PEM group (ps < 0.05). Using a graph theoretical approach, mixed ANCOVAs showed increased segregation (ps = 0.0303 and 0.0441) and decreased integration (*p* = 0.0498) in previously malnourished participants compared to healthy controls. These results can be interpreted as a compensatory mechanism to preserve cognitive functions, that could also be related to premature or pathological brain aging. To our knowledge, this study is the first NIRS neuroimaging study revealing brain function alterations in middle adulthood following early childhood malnutrition limited to the first year of life.

## 1 Introduction

Recent reports show that childhood malnutrition still affects more than two hundred million children under 5 years of age worldwide and causes 45% of deaths in this population (UNICEF et al., 2021; Zarocostas, 2023). Moderate to severe childhood malnutrition has been associated with extensive and permanent adverse effects on cognition and behavior (for a review, see Galler et al., 2021). Reported effects include lower intelligence quotient (IQ), attention and executive deficits, conduct problems and affective and depressive symptoms (Galler et al., 2010, 2012a,b,c; Waber et al., 2014a,b). However, few studies have investigated the underlying cerebral alterations in the human brain that could explain these adverse effects.

Animal research on the effects of early malnutrition showed that perinatal malnutrition in rats is associated with neuroanatomical and neurochemical changes. These include alterations in cell number, synapses, myelinization and dopaminergic and serotoninergic neurotransmission, especially in the hippocampus and the prefrontal cortex (see Alamy and Bengelloun, 2012 for a review). Interestingly, nutritional rehabilitation has been shown to partially reverse those effects (Bengelloun, 1990; Lukoyanov and Andrade, 2000). In animal functional studies, prenatal malnutrition has also been associated with hypoactivity in the prefrontal cortex (Rosene et al., 2004; McGaughy et al., 2014; Rushmore et al., 2021). Using 2-deoxyglucose as a proxy for metabolic demand and quantifying it using radiographic methods, a recent study reported lower functional activity, clustering and local efficiency in the prefrontal cortices of previously malnourished adult rats (Rushmore et al., 2021). The authors also found less functional segregation in the brain networks of these rats as measured by a lower mean clustering coefficient. Animal studies have therefore shed some light on the association between early childhood malnutrition and subsequent brain alterations.

In humans, most studies investigating the neural impact of early malnutrition are case studies or did not include sufficient statistical analyses (see Galler et al., 2021 for a review). Nevertheless, structural studies show severe diffuse cerebral atrophy and loss of brain volume in children in the acute stage of childhood malnutrition (see Gladstone et al., 2014 for a review). Early functional studies show a persistent lower dominant frequency in the electroencephalography (EEG) of toddlers hospitalized for childhood malnutrition (see Gladstone et al., 2014 for a review). Those results were confirmed by more recent studies in school-aged malnutrition survivors who consistently found increased theta activity and decreased alpha activity, potentially associated with a neurodevelopmental delay (Bartel et al., 1979; Taboada-Crispi et al., 2018; Bringas Vega et al., 2019). In another study, Xie et al. (2019) studied the impact of faltering growth (slower rate of weight gain than expected for age and sex) in toddlers living in low-resource settings on EEG functional connectivity. Height-for-age was negatively related to EEG functional connectivity in the theta (especially for connections in the frontal lobe) and the beta frequency bands, which in turn was negatively associated with children’s cognitive functioning at 48 months. Although not explicitly investigated in the context of childhood malnutrition, these findings indicate that functional connectivity could be a sensitive tool to predict cognitive outcomes later in life following childhood malnutrition. The persistence of these brain alterations following childhood malnutrition throughout adulthood remains poorly studied.

To our knowledge, only two recent studies investigated brain function in adult malnutrition survivors (Bosch-Bayard et al., 2022; Roger et al., 2022). Bosch-Bayard et al. (2022) recently found abnormal resting-state EEG source activity when comparing childhood and adulthood EEGs longitudinally in previously malnourished and healthy individuals. Overall, irrespective of age, theta and high-alpha activity in the right superior and middle frontal gyri (plus the precentral gyrus only for the high-alpha) were higher, whereas the low-alpha activity in the bilateral visual cortices was lower in the malnutrition group compared to the control group. In the same cohort, Roger et al. (2022) also found abnormal EEG evoked potentials during an attentional task, which may be associated with the attention deficits previously reported in this cohort (Galler et al., 2012b). To our knowledge, no studies have examined the impact of childhood malnutrition on the brain hemodynamic signal and functional brain network organization in adulthood. Near infrared spectroscopy (NIRS) is a functional neuroimaging technique providing a hemodynamic signal that is becoming more widely accessible in areas where malnutrition is prevalent (Boas et al., 2014). Using NIRS functional connectivity and a graph theoretical approach, which has been shown to be a promising tool for investigating the impact of childhood malnutrition on brain network topology (Xie et al., 2019; Rushmore et al., 2021), this study aims to investigate functional brain network in adults who suffered from a single episode of malnutrition restricted to their first year of life.

## 2 Materials and methods

### 2.1 Barbados Nutrition Study

The Barbados Nutrition Study (BNS) is a 50+ year longitudinal cohort study that follows a Barbadian cohort hospitalized in the first year of life for a single episode of protein-energy malnutrition (PEM) and matched healthy controls (Ramsey, 1979; Galler et al., 1983a,b). The PEM group was recruited initially between 1967 and 1972, during the children’s hospitalization for moderate to severe PEM (Gomez et al., 1955). Inclusion criteria were: (1) they had been diagnosed on clinical exam as showing the symptoms of PEM, including significant weight loss (below 75% of expected weight of age) in the absence of edema, (2) their birth weights were > 2,500 g (to exclude those children exposed to fetal growth retardation), and (3) they had Apgar scores ≥ 8. Exclusion criteria were: (1) pre- or postnatal complications, (2) encephalopathic events during childhood and (3) further malnutrition after age one. A second set of individuals who were the same ages as the PEM and CON participants and who had unambiguous diagnoses of kwashiorkor (weight loss and edema) in the first year of life were added to the BNS in 1984 (*n* = 54). However, these individuals were not included in the current study. Control participants (CON) who met the same inclusion and exclusion criteria but had no histories of malnutrition were recruited among healthy classmates of the PEM group and were matched to the PEM group with respect to age, gender and handedness. In total, the original cohort of the BNS comprised 312 individuals, 129 PEM, 54 kwashiorkor and 129 CON participants. Following their hospitalization, the previously malnourished children were enrolled in a government program that provided subsidized foods, nutritional education, home visits, medical care, and a preschool program two to three mornings per week from the first year of life until they reached 12 years of age (Ramsey, 1979). This intervention ensured that the history of PEM was limited to the first year of life and allowed them to achieve complete catch-up in physical growth by adolescence (Galler et al., 1987).

### 2.2 Participants

The reader is referred to our companion paper for more information on the selection and characteristics of the NIRS Study participants (Bosch-Bayard et al., 2022). Briefly, in summer 2018, a subset of 100 individuals (limited to PEM and CON) from the original BNS cohort were selected for a 40-year follow-up study. There were no significant differences between participants in the 2018 study and non-participants from the BNS cohort, confirming that the 2018 subgroup was representative of the larger BNS cohort (Bosch-Bayard et al., 2022). Although some individuals were lost to follow-up, the sample size at this wave of data collection was determined by funding limitations. In the current NIRS study, fifty-five adults (from the sub-group of 100 participants) were recruited from the Barbados Nutrition Study cohort. All participants in the summer 2018 fNIRS data collection were between 45 and 51 years old when tested. Participants were excluded if they were under the influence of alcohol or drugs during data acquisition (one male participant from the PEM group was excluded for this reason) or if the data quality was not sufficient (one female participant from the CON group was excluded for this reason; see section “2.5 Functional connectivity and graph theory” for specific data quality criteria). The final NIRS sample included 53 participants: 25 PEM participants and 28 controls.

This study has been performed in accordance with the ethical standards proposed in the 1964 Declaration of Helsinki and its later amendments. All study participants provided written informed consent and were compensated for their time and travel expenses. This study was approved by the Massachusetts General Hospital IRB (IRB Protocol 2015P000329/MGH), Hôpital Sainte-Justine, the Barbados Ministry of Health and Centro de Neurociencias de Cuba’s (2017/02/17/CNEURO) Ethics’ committees.

### 2.3 Procedure

**FIGURE 1:**
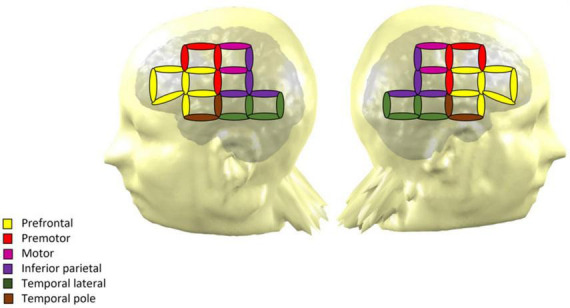
NIRS montage of the 32 optodes across the scalp over both hemispheres. The coverage yields 64 measurement channels, which are represented by ellipses. The color of each ellipse indicates the region of interest of the channel.

All participants came to the Barbados Nutrition Study Centre in Bridgetown, Barbados, for a single visit, that included a resting-state NIRS recording. Testing took place in a dimly lit and air-conditioned room. Participants were seated in a comfortable chair and fitted with an active cap with 32-NIRS optodes (16 emitters and 16 detectors yielding 64 channels with a maximum distance of 3 cm). The NIRS signal was recorded using a NIRScout device and the NIRStar Software (version 15, NIRx Medical Technologies, Glen Head, NY, USA). NIRS channels were positioned on the frontal, temporal and parietal regions (Figure 1). Regions of interest were established based on Broadman areas using a NIRS-MRI registration on an adult template (Tremblay et al., 2022). The procedure yielded seven regions of interest in each hemisphere: prefrontal cortex, Broca area, premotor cortex, motor cortex, inferior parietal cortex, lateral temporal cortex and temporal pole. Electroencephalography (EEG) recordings were performed simultaneously with the NIRS. NIRS-EEG recording was performed at rest and during a Go-No-Go attentional task, and the whole procedure lasted around 2 h. Only the resting-state NIRS data are reported in the current article. The EEG data and results from the task-based recording will be reported in subsequent publications (see also Roger et al., 2022).

The NIRS signal was recorded during a 12-min resting period. The participants were instructed to relax and look at a cross displayed on a monitor approximately 80 cm in front of them using Presentation software (version 20.2, Neurobehavioral Systems, Albany, CA, USA). They were also instructed not to fall asleep. If they were showing signs of falling asleep, the examiner would talk to them and offer them a break and a snack.

### 2.4 NIRS recording and data processing

The NIRS signal was recorded at a 7.81 Hz sampling rate. The optical intensity (DC) was acquired using two wavelengths, 760 nm and 850 nm. The optical intensity signal was preprocessed using the LIONirs toolbox (Tremblay et al., 2022), running on SPM12 (Wellcome Trust Centre for Neuroimaging, UK) in MATLAB (Version 2021b, MathWorks, Natick, MA, USA). We first performed a phase coherence-based-cardiac detection to assess the signal quality and reject noisy channels (Fourdain et al., 2023). We excluded channels with a mean phase coherence below 0.85, indicating insufficient heartbeat presence compared to the other channels and, thus, poor signal quality. We also excluded segments with motion artifacts using a standard deviation moving average and manual inspection. Channels with more than 25% bad time points were rejected. Participants with more than 50% rejected channels were excluded from further analysis (*n* = 1). We then applied a 0.01 to 0.08 Hz Butterworth digital filter to isolate the signal frequencies typically associated with spontaneous brain function and remove potential confounding signals (heartbeat 1.1 Hz, breathing 0.2 Hz, Mayer waves 0.1 Hz). The normalized optical density (i.e., the change in optical density; dOD) was computed by dividing each good segment’s signal by its average to calculate the HbO and HbR concentration changes (dCON) using the Modified Beer lambert law (Delpy et al., 1988). The differential pathlength factor (DPF) was calculated depending on the participant’s age using the method suggested by Scholkmann and Wolf (2013).

To remove the remaining physiological confounds in our frequency range of interest, we applied a principal component analysis (PCA) filter to the data using the function available in the Brain AnalyzIR toolbox (Santosa et al., 2018). More specifically, we decomposed the data from each channel using singular value decomposition and recomposed it without the first component. The physiological noise is considered the principal source of covariance between the channels in NIRS resting state data. Therefore, removing the first PCA component should extract most of this remaining noise from the signal (Santosa et al., 2020; Noah et al., 2021; Abdalmalak et al., 2022). The NIRS signal does not comply with the independency statistical assumption necessary in most statistical models because it is autocorrelated (Barker et al., 2013; Santosa et al., 2020; Abdalmalak et al., 2022; Lanka et al., 2022). To obtain more statistically valid data we thus removed the autocorrelation in the time series by applying the prewhitening function of the Brain AnalyzIR toolbox to the PCA-filtered data.

### 2.5 Functional connectivity and graph theory

We first performed functional connectivity analyses on the PCA-filtered concentration change signal using the LIONirs toolbox (Tremblay et al., 2022). We randomly selected two hundred 60-s segments in the signal. The segments were retained if at least 90% of their time points were valid. We then computed Pearson’s correlations between all channel pairs on the retained segments and averaged the resulting correlation matrices together. This method ensures that the final correlation matrix is representative of the whole 12 min-recording. A Fisher transformation was applied to the resulting matrix to normalize the correlation coefficients distribution. Finally, the Fisher-transformed correlations were averaged by regions of interest (ROI) for each participant and further used for statistical analyses.

**FIGURE 2:**
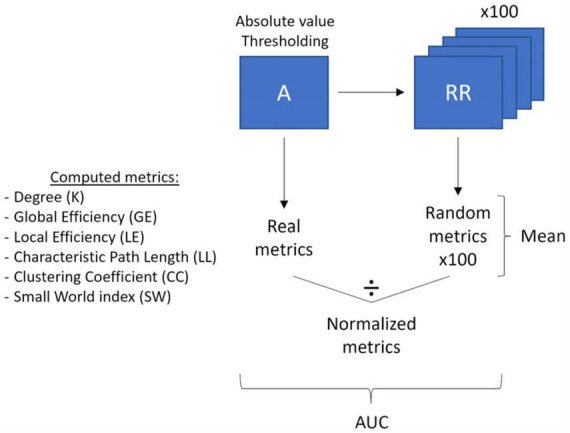
Graph theory analysis pipeline. The main processing steps (thresholding, randomization, averaging, normalization and area under the curve calculation) of the correlation matrices and the computed graph theory metrics are outlined.

We also performed graph theory analysis on the raw channel-wise Pearson’s correlation matrices using the brain connectivity toolbox (BCT; Rubinov and Sporns, 2010) and homemade scripts in MATLAB (Version R2021b, Mathworks, Inc., Natick, MA, USA; see Figure 2 for a visualization of the Graph Theory analysis pipeline). Graph Theory is a branch of mathematics used to model, estimate, and simulate the topology and dynamics of brain networks using graphs comprising nodes linked by edges. In the graph theory framework, a node is defined as a measurement channel and an edge is defined as the functional connectivity, i.e., the Pearson’s correlation, between two nodes. Graph theory allows the indirect characterization of the level of integration and segregation of a network. Integration is the ability to rapidly combine specialized information from distributed brain regions, while segregation is the ability for specialized processing to occur within densely interconnected groups of brain regions (Rubinov and Sporns, 2010). To perform graph theory analyses, the correlation matrices were first prepared. We retained the absolute values of the negative correlations as is frequently done in NIRS studies since it is still unclear how to interpret negative correlations between channels. We then applied multiple thresholds (ranging from 0.01 to 0.85 with steps of 0.01) to each participant’s correlation matrix to extract only the more relevant connections. The correlation values higher than the selected threshold were retained in the matrix, and the rest were set to 0, yielding 85 different weighted correlation matrices for each participant (“real matrices”). One hundred random matrices were computed from each weighted matrix using a randomization function and will be used to normalize the graph theory metrics in further steps. The randomization function shuffled the correlation weights while preserving the properties of the original matrix.

The graph theory metrics were then computed on each real thresholded-matrix (“real metrics”) and each of its associated 100-random matrices (“random metrics”). The following graph theory metrics were used: degree, global efficiency, local efficiency, characteristic path length, clustering coefficient, and small-world index. The degree (K) refers to the number of nodes connected to each node in the matrix (i.e., the number of neighbors of each node). The global efficiency (GE) is the average of the inverse shortest distance between all pairs of nodes in a network and represents how easily and effectively the network communicates information. The local efficiency (LE) is similar to the global efficiency but is computed on the node scale instead of the whole matrix. It refers to how effectively the neighbors of a node communicate with each other once this node has been removed. The characteristic path length (LL) is the average shortest distance between all pairs of nodes in the network. The clustering coefficient (CC) is the fraction of a node’s neighbors that are also neighbors of each other and relates to the tendency of neighbors of a node to cluster together. To reduce the data dimensionality, the nodes average of LE and CC was computed for each participant. For each previously presented metric, we normalized each participant’s real metric by dividing it by the mean of all participants’ random metrics, yielding a set of normalized graph theory metrics (“norm metrics”). Finally, the small-world index (SW) was computed by dividing the normalized clustering coefficient (NormCC) by the normalized characteristic path length (NormLL). It reflects the balance between network integration and segregation in comparison to random networks. For more information on graph theory metrics, see Rubinov and Sporns (2010) or Fornito et al. (2016).

In order to isolate the most relevant thresholds to characterize the functional brain networks differences between our groups, we restricted our analyses to a subrange of thresholds selected according to Bassett et al.’ 2008 criteria. Indeed, using the 0.01 to 0.85 thresholded real metrics, we identified the thresholds where the average matrices of our participants were fully connected (Kmin ≥ 1 and CCmin ≥ 0) and could be described as organized in a small-world topology (SW ≥ 1), according to what is usually expected for the brain organization. Lastly, to reduce the number of statistical comparisons, the area under the curve (AUC) of the normalized metrics values in the selected range of thresholds was computed and subsequently used for statistical analyses.

### 2.6 Covariates

A socioeconomic (SES) index measure was collected in the first BNS data collection when the participants were 5–11 years of age using the Ecology Questionnaire (Galler and Ramsey, 1985; Galler et al., 2012c). The questionnaire completed by parents/primary caretakers included items about conditions in the home and parental educational level, and employment history. This measure was used in the current study to control for initial childhood socioeconomic discrepancies between our two groups. Principal component analysis using varimax rotation was computed on the questionnaire data and yielded a household standard of living factor, which was used in the current study as a childhood SES measure (see Galler and Ramsey, 1985 for details and psychometric properties). We adjusted for childhood socioeconomic status but not adult socioeconomic status, because the latter was viewed as a potential outcome of longstanding functional compromise associated with the early malnutrition (Galler et al., 2012c). Age and sex were also considered as covariates, but were not included in the final analyses since there were no difference between PEM and control groups in the mean age or percent of males and females of the study participants.

### 2.7 Statistical analysis

Statistical analyses were performed using R (Version 4.2.2, R Foundation for Statistical Computing, Vienna, Austria) and SPSS (Version 28.0.1.0, IBM SPSS Statistics, Armonk, NY, USA). The significant *p*-value was set to *p* ≤ 0.05. Group differences in ROI averaged correlations were tested using permutation analysis (*boot* R package) and mixed ANCOVAs (*car* R package), with the Group as a between-subject factor and the SES as a covariate. More specifically, the F values distribution is computed by randomly permuting 2,000 times the cases in the data. The true F value can then be compared to the F value distribution to test for a significant effect. This procedure was done for each ROI pair to test for Group differences. A false discovery rate correction for multiple comparisons was also applied to the resulting *p*-values. Group differences in normalized graph theory metrics’ AUC were also tested using ANCOVAs with the Group as a between-subject factor and SES as a covariate.

## 3 Results

### 3.1 Demographic characteristics

The demographic characteristics of the sample are reported in Table 1. The two groups (PEM vs. Control) did not differ in age, gender, or handedness. However, there were significant differences in childhood standard of living, indicating lower SES (childhood ecology factor scores) in the PEM vs. Control groups. SES was therefore controlled in all statistical analyses. No statistical differences (*p* > 0.05) were present between nutrition groups for medical comorbidities (e.g., diabetes, hypertension, encephalopathic events, alcoholism or cannabis abuse). These conditions were therefore not controlled in further statistical analyses.

**TABLE 1:** Demographic characteristics of participants.

Characteristic	PEM	Control	*t*-test/χ 2	*p*
*N*	25	28		
Males [*N* (%)]	14 (56.00)	14 (50.00)	0.19	0.66
Age (years)	48.70 (1.89)	48.54 (1.93)	−0.30	0.39
Age range (years)	45.39–51.55	45.50–51.37		
Handedness [*N* left (%)]	3 (12.00)	3 (10.70)		0.61
Childhood ecology factor	−1.13 (0.77)	−0.32 (0.75)	3.93	< 0.001

Handedness statistical tests were done using a Fisher exact test (non-parametric).

### 3.2 Functional connectivity

**FIGURE 3:**
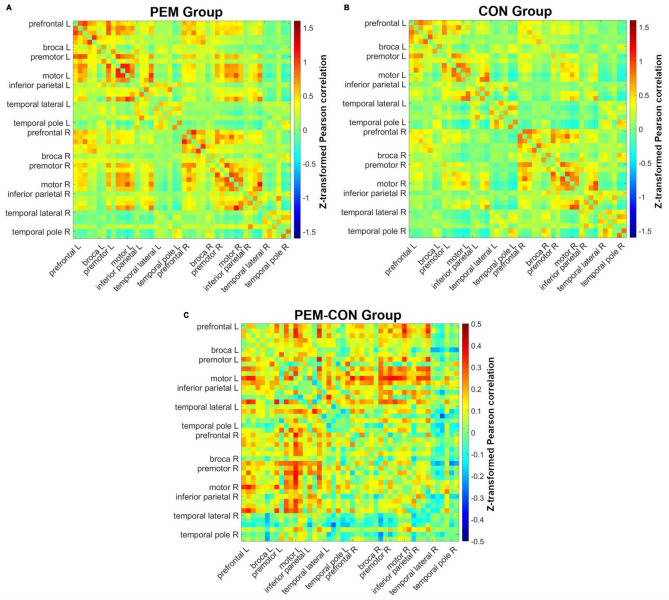
Pearson correlation matrices for each channel pair. Hotter colors indicate stronger correlation strength, whereas colder colors indicate weaker correlation strength. Channels are ordered from the left anterior to the right posterior location (left anterior, left posterior, right anterior, right posterior). **(A)** Mean correlations of all channels for the PEM group. **(B)** Mean correlations of all channels for the CON group. **(C)** Difference in mean correlations of all channels between the PEM and CON groups.

**FIGURE 4:**
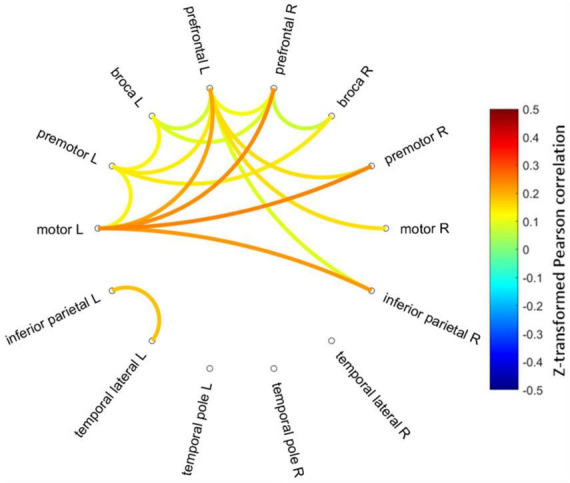
Connectogram of the significantly different inter-ROIs Pearson correlations between the PEM and CON groups (PEM-CON; uncorrected). Hotter colors indicate larger correlation strength difference, whereas colder colors indicate smaller correlation strength difference between the groups. Regions are ordered from more anterior locations at the top to more posterior locations at the bottom, with the left hemisphere regions on the left side of the connectogram and right hemisphere regions on the right side of the connectogram.

Figure 3 shows mean Fisher transformed Pearson’s correlation for each group (A: PEM; B: Control), and group differences (C: PEM—CON). Applying FDR to correct for multiple comparisons yielded no significant effect. Therefore, uncorrected group differences are briefly presented. SES-corrected ANCOVAs show stronger correlations in the frontal region for the PEM group compared to the CON group (see Figure 4). More specifically, there are stronger interhemispheric connections between the left and right frontal regions in the PEM group compared to the Control group and, to a lesser extent, within the left frontal areas. No significant group differences, except for one connection (right prefrontal—right Broca areas), can be found within the right hemisphere (see Table 2 for the details).

**TABLE 2:** Significantly different functional connections between the groups (uncorrected).

	F	uncorrected *p*
**Intrahemispheric L**		
PrefrontalL-PremotorL	4.8216	0.0340
PrefrontalL-BrocaL	3.9370	0.0450
BrocaL-PremotorL	4.3220	0.0400
PremotorL-MotorL	5.0782	0.0360
MotorL-PrefrontalL	7.6391	0.0090
Inferior ParietalL-Temporal lateralL	7.1867	0.0100
**Interhemispheric**		
PrefrontalL-PrefrontalR	6.4137	0.0115
PrefrontalL-PremotorR	4.4980	0.0390
PrefrontalL-MotorR	5.3021	0.0300
PrefrontalL-Inferior ParietalR	5.7337	0.0225
BrocaL-PrefrontalR	4.1724	0.0345
PremotorL-BrocaR	4.6697	0.0390
MotorL-PrefrontalR	5.4037	0.0215
MotorL-PremotorR	4.9457	0.0300
MotorL-Inferior ParietalR	9.4563	0.0045
**Intrahemispheric R**		
PrefrontalR-BrocaR	5.3372	0.0260

### 3.3 Graph theory

**FIGURE 5:**
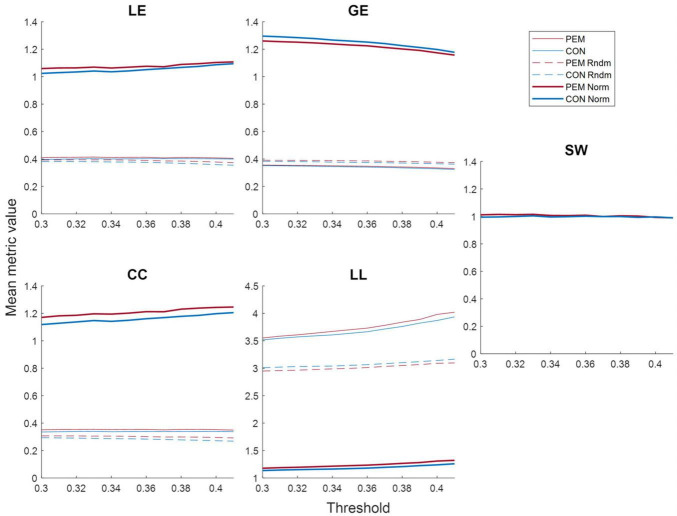
Mean graph theory metrics values (real, random and normalized) for each group according to the threshold: local efficiency (LE), global efficiency (GE), clustering coefficient (CC), characteristic path length (LL) and small-world index (SW). The values for the PEM group are displayed in red, while the values for the CON group are displayed in blue. Solid lines represent real values, dashed lines represent random values, and bold lines represent normalized values.

Figure 5 shows the mean metrics values in function of the threshold for each group. The area under the curve of the normalized metrics in the selected threshold range (0.30–0.41) was computed and analyzed. ANCOVAs revealed a significant Group effect for the clustering coefficient [CC; *F*(49) = 4.2709, *p* = 0.0441], the local efficiency [LE; *F*(49) = 4.9741, *p* = 0.0303] and the global efficiency [GE; *F*(49) = 4.0467, *p* = 0.0498]. The CC and the LE AUC values were higher for the PEM group than the CON group, whereas the GE AUC values were higher for the CON group than the PEM group. The characteristic path length [LL; *F*(49) = 0.3420, *p* = 0.5614] and the small-world index [SW; *F*(49) = 3.5382, *p* = 0.0659], however, yielded no significant Group effect. Graph theory results are summarized in Table 3.

**TABLE 3:** Significantly different graph theory metrics’ AUC values between the groups.

	F	corrected *p*	Group difference
Clustering coefficient (CC)	4.2709	0.0441	PEM > CON
Local efficiency (LE)	4.9741	0.0303	PEM > CON
Global efficiency (GE)	4.0467	0.0498	CON > PEM
Characteristic path length (LL)	0.3420	0.5614	
Small-world network index (SW)	3.5382	0.0659	

## 4 Discussion

This study aimed to explore the impact of moderate to severe protein-energy malnutrition in the first year of life on brain function at 45–51 years of age using NIRS functional connectivity analyses and a graph theoretical approach. Results show stronger frontal correlations in the PEM group compared to the CON group when controlling for SES, but not for multiplicity of comparisons. Furthermore, graph theory analyses reveal a higher clustering coefficient, local efficiency, and lower global efficiency in childhood malnutrition survivors compared to controls. Overall, these results highlight abnormal functional brain connectivity following early childhood malnutrition, suggesting long-lasting functional cerebral alterations following a single episode of childhood malnutrition restricted to the first year of life.

### 4.1 Cerebral functional connectivity and malnutrition

The long-term impact of malnutrition on functional brain connectivity, notably in frontal areas, has been previously reported in animal and human studies. Indeed, prefrontal functional alterations have been found at rest (McGaughy et al., 2014) and during an attention task (Rushmore et al., 2021) in prenatally malnourished adult rats using the 2-deoxyglucose (2DG) metabolic marker. Alterations in resting-state and task-based EEG activity have also been consistently reported in children and adults with histories of malnutrition in early childhood (Bartel et al., 1979; Taboada-Crispi et al., 2018; Bringas Vega et al., 2019; Bosch-Bayard et al., 2022; Roger et al., 2022). A recent study examined the effects of famine exposure during gestation on resting-state brain functional connectivity using fMRI in 68 years old adults from the Dutch Famine Study (Boots et al., 2022). Exposed participants had lower connectivity within the default mode network (DMN; posterior cingulate cortex, medial prefrontal cortex, medial temporal lobe, angular gyrus), within the salience network (SN; frontoinsular cortex, dorsal anterior cingulate cortex), and between the DMN and the central executive network (CEN; dorsolateral prefrontal cortex, lateral posterior parietal cortex), as well as higher connectivity within the CEN as compared with unexposed adults. Although the timing of the nutritional insult was different in the BNS and Dutch Famine studies, the hyperconnectivity in the CEN found by Boots et al. (2022), being the sole network involving only cortical regions, aligns with the frontal hyperconnectivity we report in the current study using fNIRS (measures only cortical and not subcortical structures). Overall, our results in postnatally malnourished adults are in concordance with the literature, showing a pattern of heightened cortical functional connectivity following childhood malnutrition, with a specific vulnerability present in the frontal cortex.

Hyperconnectivity in various brain regions and networks has also been reported in other clinical populations. For instance, adults with mild cognitive impairment (MCI) or Alzheimer’s disease (AD) show patterns of hypo- and hyperconnectivity within the DMN, as well as hyperconnectivity within the SN and the limbic network (Badhwar et al., 2017), while typically aging adults show a decrease in connectivity within the nodes of several resting state networks, such as the DMN, the SN and the attention/executive network (Sala-Llonch et al., 2015). The increased connectivity in pathological aging has been interpreted as a compensatory mechanism in response to neurodegeneration. According to this compensation hypothesis (Park and Reuter-Lorenz, 2009), elderly individuals with MCI or AD demonstrate an increase in brain activity to compensate for declining neural structure and function. In the current study, the hyperconnectivity found in previously malnourished adults could thus be a compensatory mechanism for the impact of malnutrition on the brain. Subsequent follow-up of this cohort will be necessary to confirm this hypothesis.

### 4.2 Functional brain network topology and malnutrition

Our graph theory results revealed differences in functional brain network organization in the PEM group compared to controls suggesting long-lasting impaired brain function organization following childhood malnutrition. More specifically, we found in PEM participants a higher clustering coefficient and local efficiency, indicating an increased segregation, and lower global efficiency indicating a decreased integration. Those results can thus be interpreted as a disruption in the integration and the segregation of the brain networks. To our knowledge, no other human malnutrition study previously used graph theory measures. However, findings from healthy aging studies can provide relevant information for interpretation. These studies highlight an age-related decrease in long-range connectivity (i.e., integration) and an increase in short-range connectivity (i.e., segregation; Toussaint et al., 2011; Tomasi and Volkow, 2012; Ferreira and Busatto, 2013; Cao et al., 2014; Sala-Llonch et al., 2014). These findings are in line with the results of the current study as brain organization resembling that of older adults may indicate premature brain aging following childhood malnutrition. This hypothesis was previously put forward by another research group studying the impact of prenatal famine exposure, who showed advanced brain aging by about 4 years in the exposed participants compared to controls (Franke et al., 2018). The hypothesis of premature brain aging would also be consistent with the accelerated cognitive decline reported in our cohort (Razzaq et al., 2020). More studies in adult malnutrition survivors and follow up neuroimaging in this cohort will be needed to determine if childhood malnutrition is associated with accelerated aging. Eventually, this body of literature may lead to the elaboration of artificial intelligence prognostic and diagnostic models of early childhood exposures to improve long-term brain, cognitive and behavioral outcomes and elaborate more targeted interventions strategies (Murugesan et al., 2020; Mofatteh, 2021). These findings could also contribute to the conceptualization of a cumulative risk model of disease progression applicable to vulnerable populations in low- and middle-income settings.

### 4.3 Limitations

This study has several limitations that must be considered when interpreting its results. First, the sample size is modest, and could have prevented us from revealing other significant effects due to a lack of statistical power or selection bias. We found no evidence of bias between individuals who were included (21% of the original cohort of PEM subjects) and those who did not participate in the current study in terms of age, gender, and nutrition group membership or childhood standard of living (Bosch-Bayard et al., 2022), suggesting that our participants were representative of the BNS cohort. However, there could have been some unknown source of bias that we did not detect, potentially compromising generalizability. Second, considering the absence of significant correlation difference between the groups using multiple comparison corrections, the results of the functional connectivity analysis without applying such corrections need to be interpreted cautiously and further replicated. Third, although we used the best available data to control experimentally for socioeconomic (SES) differences between previously malnourished participants and controls, we cannot be certain that group differences were fully attributable to the history of early malnutrition and that there were not undetected experiential or genetic differences between the groups. Notably, malnutrition is a multi-dimensional condition with co-occurring adversity factors such as poverty and child maltreatment (Hock et al., 2020) that may play a role in the results presented in this study. Finally, further longitudinal studies are also needed to better understand the evolution of those alterations over time and as the participants grow older. Overall, future neuroimaging malnutrition studies should thus be undertaken with a larger sample size and a longitudinal design.

## 5 Conclusion

This study presents evidence of functional brain alterations in 45–51 years old adults after experiencing childhood malnutrition limited to the first year of life. They demonstrate patterns of hyperconnectivity, increased segregation and decreased integration compared to healthy controls with a specific vulnerability of the frontal cortex. These results can be interpreted as a compensatory mechanism to preserve cognitive functions. This is one of the first studies to investigate long-term brain function alterations using NIRS, a functional hemodynamic brain imaging technique.

## Data availability statement

The data that supports the findings of this study is available from the corresponding author AG, upon reasonable request.

## Ethics statement

The studies involving humans were approved by the Massachusetts General Hospital IRB, CHU Sainte-Justine, Centro de Neurociencias de Cuba, and Barbados Ministry of Health’s Ethics Committees. Current oversight is provided by the Massachusetts General Brigham IRB (Protocol No. 2015P000329). The studies were conducted in accordance with the local legislation and institutional requirements. The participants provided their written informed consent to participate in this study.

## Author contributions

KR: Data curation, Formal analysis, Investigation, Methodology, Software, Validation, Visualization, Writing – original draft, Writing – review & editing. PV: Investigation, Methodology, Project administration, Supervision, Validation, Writing – review & editing. JT: Methodology, Software, Supervision, Validation, Visualization, Writing – review & editing. MB: Conceptualization, Funding acquisition, Methodology, Resources, Validation, Writing – review & editing. CB: Conceptualization, Methodology, Project administration, Writing – review & editing. AR: Conceptualization, Methodology, Project administration, Writing – review & editing. PV-S: Conceptualization, Funding acquisition, Methodology, Resources, Software, Supervision, Validation, Writing – review & editing. JG: Conceptualization, Funding acquisition, Methodology, Project administration, Resources, Supervision, Validation, Writing – review & editing. AG: Project administration, Resources, Supervision, Validation, Writing – review & editing, Conceptualization, Funding acquisition, Methodology.
